# Content Analysis of Warning Signs Identified as Part of Crisis Response Planning in a Community Sample of Gun Owners and Non-owners

**DOI:** 10.3389/fpsyt.2022.867332

**Published:** 2022-04-21

**Authors:** Christina Rose Bauder, Jarrod M. Hay, James G. McClung, Austin G. Starkey, Craig J. Bryan

**Affiliations:** Department of Psychiatry and Behavioral Health, The Ohio State University College of Medicine, Columbus, OH, United States

**Keywords:** suicide, suicide-specific interventions, warning signs, crisis response planning, firearms, content analysis

## Abstract

**Background:**

Assessing for and identifying those at imminent risk for suicide continues to present challenges, especially as many who die do not interact with specialty mental health treatment preceding suicide. Suicide-specific interventions in healthcare settings have been found to improve suicide-related outcomes, yet little is known about the confluence of behavioral, cognitive, emotional, and physiological indicators of emotional distress as they correspond to other key risk characteristics and high-risk groups like gun owners.

**Aim:**

The purpose of this content analysis was to examine self-identified warning signs of distress between gun owners and non-owners through crisis response planning (CRP).

**Methods:**

Participants completed a collaborative CRP. Warning signs were categorized as being either behavioral, cognitive, emotional, or physiological in nature. Bivariate logistic regression models were used to examine associations between firearm ownership and variables of interest. Participants were evenly split between men (*n* = 44) and women (*n* = 44) and were predominantly white (67.1%) with a mean age of 35.9 (*SD* = 13.6).

**Results:**

Emotional warning signs of distress (68.2%) were reported slightly more often than behavioral (65.9%) followed by physiological (52.3%), and cognitive (46.6%). Firearm owners were significantly more likely to be male (*OR* = 2.5, 95%CI [1.07–6.0]). All participants were about a fourth as likely to report both a behavioral and physiological warning sign concurrently (*OR* = 0.26, 95% CI [0.09–0.67]).

**Conclusion:**

Similarities and departures in warning signs of emotional distress may inform future research exploring both self-reported warning signs and related self-management strategies identified through suicide-specific interventions, particularly among high-risk groups such as gun owners.

## Introduction

Suicide remains a leading cause of death in the US with the rate of suicide increasing by more than 30% since 1999 (1). Most deaths are enacted by firearms, accounting for more than half of suicide deaths in 2020 (2). Despite improved research in identification and treatment for those at risk, fewer than half of suicide decedents are engaged with specialty mental health treatment in the months preceding their deaths (3, 4). Emergency departments and primary care clinics, by comparison, are much more likely to be visited (5). Brief suicide prevention interventions that are effective and feasible in these settings have therefore been developed. The crisis response plan [CRP; (6)] and the related safety planning intervention [SPI; (7)] are two such strategies. The CRP and SPI share critical key components including identification of warning signs, self-management strategies, sources of support (e.g., family, friends, or loved ones), and emergency resources. Identifying personal warning signs of an emerging crisis is considered a critical first component of the CRP and SPI because it helps patients to better recognize when they are shifting to a higher risk state (8, 9). Warning signs are therefore intended to differentiate proximal factors that indicate imminent or near-term suicide risk, as compared to risk factors that are more distally related to suicide (e.g., demographic or historical variables) (8).

Research indicates emotion regulation is impaired among individuals with increased suicide risk, though less in known as to whether these individuals can aptly recognize dysregulation preceding a suicide crisis (10–14). For example, suicidal people are more likely to use maladaptive strategies like rumination and are less likely to use adaptive strategies like reappraisal (12, 15–19). These deficits increase the likelihood of experiencing acute suicidal episodes in response to stressful events. Given the lethality of suicidal acts by firearms (90%) in comparison to other means, consideration of brief suicide prevention interventions among gun owners is timely (20). Strengthening a person's capacity to identify their personal warning signs could therefore avert suicidal behavior during an acute crisis.

Although warning signs are assumed to be an especially critical component of brief suicide prevention interventions, they have received only limited empirical attention. Variations in warning signs between gun owners and non-owners have not been examined, therefore, very little in known as to similarities and departures between these groups. Such research could reveal valuable information about the phenomenology of acute suicidal crises. Exploration of suicide-related phenomena has employed various qualitative approaches, including content analysis [see (21–23)], yet this approach has not been used in examining warning signs of a suicide crisis. To achieve this objective, we used content analysis (24) to compile and describe patterns of warning signs generated as part of a collaborative CRP and explored differences in these patterns between participants based on age, sex, and race and among those with and without a history of suicidal thoughts and behaviors. We additionally examined reported warning signs between those who do and do not own firearms for potential differences. Combining qualitative and quantitative data analysis capitalizes on the strengths of each methodology and allows for triangulation of the data to better understand the complex processes associated with complex phenomena, such as suicide (25–27).

### Crisis Response Planning

The CRP is a brief intervention in which a clinician and an individual collaboratively identify personal indicators or warning signs of an emotional crisis, self-management strategies, reasons for living, sources of social support, and sources of professional and crisis support (6, 28). Several clinical trials support the efficacy of the CRP and SPI for reducing suicide attempts (29) and other suicidal behaviors (30) as compared to treatment as usual.

Typically, these suicide-focused interventions are administered to individuals with a history of suicide attempts or ideation, however, there is consideration that completing a CRP may be a prevention tool in the development of suicidal thoughts and behaviors in a non-clinical sample. Preliminary research suggests the CRP may lead to short-term increases in positive emotional states including hope (28) and optimism (31) when the intervention was enhanced with a brief discussion about suicidal persons' reasons for living. The CRP may also serve individuals in creating a plan to navigate and reduce the impact of emotional distress based on self-reported warning signs that may or may not be suicide specific. As the majority of suicide decedents do not access mental health treatment or care, especially those who own and have access to firearms, the CRP may provide opportunities to impart self-management strategies among a high-risk group in non-clinical settings.

There are three aims of this study; first, to compile warning signs from participant CRPs from a community sample; second, to examine and categorize reported warning signs as they correspond to the suicidal mode (32) and analyze associations based on other key participant characteristics, including suicidal ideation; and third, to examine potential themes and departures between gun owners and non-owners.

## Methods

### Participants and Procedures

Participants included 88 adults enrolled in a study aimed at exploring the mechanisms underlying vulnerability to firearm suicide. Inclusion criteria for the parent study were ≥18 years old and having regular access to either an Android or Apple smartphone, and the exclusion criteria were serious medical conditions that would interfere with data collection procedures (e.g., deafness, moderate or severe traumatic brain injury, lifetime mania or psychosis), psychotropic medication use within the past 4 months, acute alcohol intoxication (verified via breath test), and heavy recreational alcohol or cannabis use (defined as 5+ alcohol binges per month and cannabis use more than 5 times per week).

**Figure 1:**
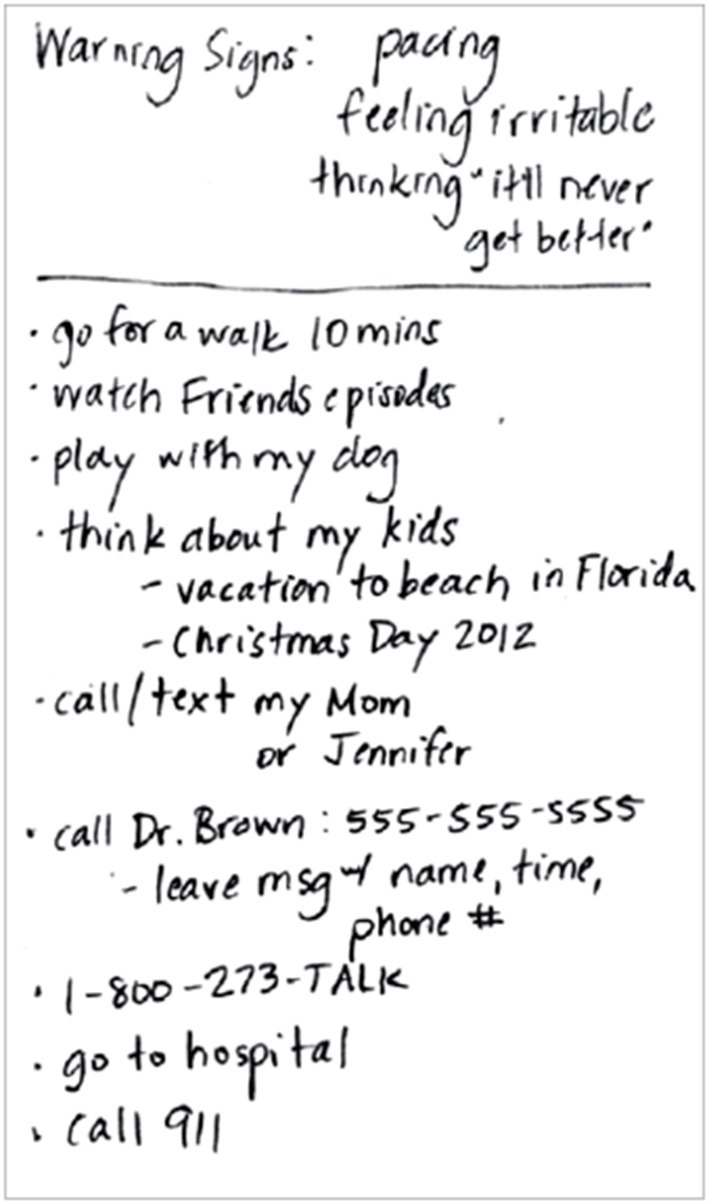
Example crisis response plan (9).

As part of the study's safety protocol, after completing within-lab study procedures, a trained member of the research team collaboratively developed a CRP with each participant lasting between 20 and 60 min. CRPs were led by either trained research personnel or masters and doctoral level clinicians. The team member first conducted a narrative assessment of a recent emotional crisis. Participants reporting a history of suicidal thoughts and behaviors were directed to “tell the story” of their most recent suicidal episode or suicide attempt, whereas those without a history of suicidal thoughts and behaviors were directed to instead “tell the story” of a recent time during which they felt very stressed or overwhelmed. The purpose of the narrative assessment is to increase understanding of the person's situation with prompting questions such as: “Would you mind sharing with me the story of what happened [today]?” (8). The narrative assessment also allows for contextualizing the chain of events leading up to the crisis. Questions such as, “What happened next?” and, “When that happened, what did you say to yourself?” may be asked to obtain more information around the chain of events. An example of completed CRP is presented in Figure 1.

After completing the narrative assessment, the team member then explained the purpose of the CRP and prompted participants to identify their personal warning signs. The remaining four components of the CRP—self-management strategies, reasons for living, sources of social support, and professional resources and crisis services—were then completed but are not the focus of the present analysis. The CRP was handwritten by the participant on an index card provided during the lab visit. Copies of each CRP, which were text recorded verbatim by the administrator, were then saved on a secure electronic storage system. CRP responses were subsequently compiled by two research personnel. Two independent raters then independently transferred responses to prepare for analysis. All subjects gave written informed consent in accordance with the Declaration of Helsinki. The study was reviewed and approved by The Ohio State University's Institutional Review Board.

### Measures

#### Warning Signs

Participants' warning signs of emotional crisis were categorized through directed content analysis as either behavioral, cognitive, physical, and emotional (24, 32, 33). Participants who indicated at least one warning sign that, based on the initial theme and alignment within the suicidal mode (33), fit into one of the four discrete categories were considered to positively endorse that item. For example, if an individual wrote, “heart beating hard” on the CRP, it was first coded as “chest issues” and then “physiological” as it corresponded to the suicidal mode. Additional examples illustrating the process of coding responses using directed content analysis are reported in Table 1.

**Table 1:** Content, code, and categories of warning signs from participant crisis response plans.

**Example CRP Content**	**Code**	**Category**
Heart beating hard	Stomach/Chest Issues	Physiological
Clenching my fists	Tense	Behavioral
Questioning myself, “What am	Disruption of Thought	Cognitive
I doing here?”		
Pacing	Restlessness	Behavioral
Arguing with my girlfriend	Engaging in conflict	Behavioral
Angry	Anger	Emotional
Irritable	Irritability	Emotional
Not sleeping	Sleep Issues	Behavioral
Hands sweat a lot	Change in perspiration	Physiological
Ruminate on bad events	Pessimistic thoughts	Cognitive
Feeling like stepping away	Withdrawal	Behavioral
Poor eating or no appetite	Dietary Changes	Behavioral
Nervousness	Anxiety	Emotional
Dry Mouth	Dry Mouth	Physiological
Fatigue	Fatigue	Physiological
Mind fog	Disruption of Thought	Cognitive
Feel impotent	Sadness	Emotional

#### Self-Injurious Thoughts and Behaviors Interview—Revised

Participants were administered the Self-Injurious Thoughts and Behaviors Interview-Revised [SITBI-R; (34)] by research personnel who were trained and supervised by a licensed clinical psychologist. Items on the SITBI-R assess for a range of self-injurious thoughts and behaviors, including suicidal ideation, suicide planning, non-suicidal self-injury (NSSI), aborted suicide attempts, interrupted attempts, and actual suicide attempts. The SITBI-R's validity has been established (34).

### Data Analysis

Qualitative data, warning sign responses, were copied verbatim to facilitate directed content analysis in three steps (24, 33), CRPs were first coded by generating preliminary themes across warning signs, such as “stomach/chest issues,” “angry,” “restlessness” and “fatigue.” Then these preliminary themes were then compared to the four established constructs of suicidal mode, a conceptual model of suicide used in brief cognitive behavioral therapy for suicide prevention (BCBT-SP) and then categorized as behavioral, cognitive, emotional, and physical by two independent raters (9, 32). Lastly, all categorized responses were then verified by the primary author for both consistency in translation of coding themes and for reflexivity. The independently coded responses were reviewed by the primary author who validated the final coding scheme based on suggested standards for reliability in content analysis (24). All CRPs were analyzed for interrater reliability using Krippendorff's alpha (35, 36). Krippendorff's alpha (35) is considered the standard reliability statistic for content analysis and related analyses. Acceptable reliability using Krippendorff's alpha statistic in social science often requires α ≥ 0.80 with the “lowest conceivable limit” set to α ≥ 0.667 for exploratory analyses [(24), p. 354]. There was sufficient reliability between raters for behavioral (0.90), cognitive (1.00), emotional (0.97) and physiological (1.00) warning signs.

All statistical analyses were performed using StataIC 16 (37). Sample descriptive statistics based on gun ownership are reported in Table 2. A series of chi-square (χ^2^) analyses were conducted to assess for the potentially confounding relationship between warning signs. Bivariate logistic regression models were used to examine participant characteristics between those who owned a firearm and those who did not. This first series of analyses examined whether firearm ownership was associated differently based on participants' age, sex, and race. Second, we examined whether firearm ownership was significantly associated with specific warning signs. We used McFadden's multinomial model estimation to determine model fit (38).

**Table 2:** Sample characteristics of non-owners and gun owners (*N* = 88).

**Variable**	**n (%)/M (SD)**	**Non-owners**	**Gun Owners**
Sex, n (%)			
Male	44 (50.0)	19	25
Female	44 (50.0)	29	15
Race, n (%)			
White	59 (67.1)	33	26
Black or African American	16 (18.2)	8	6
Other	13 (14.8)	7	8
Age, M (SD)			
	35.9 (13.6)		
Warning Signs Endorsed, n (%)			
Behavioral	58 (65.9)	28	30
Cognitive	41 (46.6)	24	17
Emotional	60 (68.2)	30	30
Physiological	46 (52.3)	28	18
SITBI-R			
Passive Suicidal Ideation	33 (37.5)	16	17
Active Suicidal Ideation	23 (26.1)	9	14
Both	22 (25.0)	9	13

## Results

Participants were split evenly between males (*n* = 44, 50%) and females (*n* = 44, 50%) and ranged in age from 19 to 70 years old (*M* = 35.9, *S.D*. = 13.5; reported in Table 2). Racial distribution was predominately White (67.05%; *n* = 59), with 18.2% identifying as Black or African American (*n* = 16), and fewer than 15% (14.8%, *n* = 13) identifying as Native American Alaskan Native, Asian, Native Hawaiian or other Pacific Islander, or multiracial.

Two thirds (65.9%) of participants endorsed at least one behavioral warning sign, nearly half of participants (46.6%, *n* = 41) endorsed at least one cognitive warning sign, 68.2% (*n* = 60) endorsed emotional warning signs, and 52.3% (*n* = 46) endorsed at least one physiological warning sign. 45.5% of participants owned at least one firearm. 37.5% of participants reported past suicidal ideation, with 26.1% reporting current suicidal ideation; a quarter of participants (25%) reported both passive and active suicidal ideation.

### Univariate Analyses of Warning Signs

There was no significant relationship between behavioral and cognitive (χ^2^ = 0.21, p = 0.65) or emotional (χ*2* = 0.56, *p* = 0.46) warning signs, or between cognitive and emotional (χ^2^ = 0.19, *p* = 0.47) or physiological (χ^2^ = 2.33, *p* = 0.13) warning signs. There was a significant association between reports of behavioral and physiological (χ^2^ = 5.73, *p* < 0.01) warning signs; participants were a quarter as likely to report both a physiological and behavioral warning sign (*OR* = 0.26, 95% CI [0.09–0.67]).

### Key Characteristics as Predictors of Warning Signs

There were no significant relationships between age and reports of behavioral (*OR* = 0.98, 95% CI [0.95–1.02]), cognitive (*OR* = 0.99 95% CI [0.96–1.02]) emotional (*OR* = 1.02, 95% CI [0.99–1.06]), or physiological warning signs (*OR* = 1.00, 95% CI [0.96–1.03]). There were also no significant differences between male and female participants: behavioral (*OR* = 0.78, 95% CI [0.29–2.06]), cognitive (*OR* = 1.58, 95% CI [0.29–3.68]), emotional (*OR* = 0.73, 95% CI [0.29–1.80]), or physiological (*OR* = 0.91, 95% CI [0.39–2.12]). Lastly, multinomial logistic regressions were performed to create a model of the relationship between warning signs and participants who identified as Black or African American or Other when compared to participants who identified as White. The final models using race as a predictor for behavioral (χ^2^ =2.01, McFadden's R^2^ =0.02, p =0.37), cognitive (χ^2^ =3.97, McFadden's R^2^ =0.03, *p* = 0.13), emotional (χ^2^ = 2.20, McFadden's R^2^ = 0.02, *p* = 0.33), and physiological (χ^2^ = 3.82, McFadden's R^2^ = 0.03, *p* = 0.14) warning signs were all not found to be significant predictors.

### Participant Characteristics, Warning Signs, and Suicidal Ideation Compared Between Gun Owners and Non-owners

Our results of the multivariate logistic regression analyses of participant characteristics, warning signs, and suicidal ideation between gun owners and non-owners are reported in Table 3. There was no significant difference between gun owners and non-owners in reporting behavioral (*OR* = 2.14, 95% CI [0.86–5.36], *p* = 0.10), cognitive (*OR* = 0.74, 95% CI [0.32–1.72], *p* = 0.48), emotional, (*OR* = 2.07, 95% CI [0.80–5.31], *p* = 0.13), or physiological (*OR* = 0.97, 95% CI [0.41–2.26], *p* = 0.94) warning signs of distress.

**Table 3:** Results of multivariate logistic regressions of participant characteristics, warning signs, and suicidal ideation between gun owners and non-owners.

	**OR**	**95% CI**	**SE**	***p*-value**
Age	0.97	(0.95–1.01)	0.01	0.16
Sex				
Male	2.54	(1.07–6.02)	1.12	0.03
Race				
Black or African American	1.27	(0.42–3.84)	0.72	0.67
Other	1.09	(0.32–3.63)	0.67	0.89
Warning Signs				
Behavioral	2.14	(0.86–5.36)	1.00	0.10
Cognitive	0.74	(0.32–1.72)	0.32	0.48
Emotional	1.80	(0.72–4.53)	0.85	0.21
Physiological	0.58	(0.25–1.36)	0.25	0.21
SITBI-R				
Passive Suicidal Ideation	1.48	(0.62–3.52)	0.65	0.38
Active Suicidal Ideation	2.34	(0.88–6.18)	1.16	0.08
Both	2.08	(0.78–5.57)	1.04	0.14

*For all analyses the reference categories for other categorical variables are as follows: Female (sex), White (race) CI, confidence interval; OR, odds ratio; SE, standard error*.

## Discussion

There were three aims of this study; first, to categorize self-reported warning signs of emotional distress from crisis response plans completed among a community sample using content analysis; second, to examine these warning signs coded as behavioral, cognitive, emotional, and physiological, in relation to other key characteristics, including suicidal ideation and third; to examine potential associations between warning signs among gun owners and non-owners. To our knowledge, this study is the first to categorize responses to brief suicide prevention interventions, like the CRP or SPI, and consider associations with gun ownership status. The findings of our study reveal the novelty of categorizing warning signs of distress and examining differences in characteristics between gun and non-gun owners.

First, we had strong interrater reliability between the two independent ratings of the four warning signs from CRPs. Our interrater reliability, calculated using Krippendorff's alpha (35), serves to also inform the face validity of our approach, specifically directive content analysis as guided by the suicidal mode (32). Second, most participants reported at least one behavioral warning sign, such as pacing, arguing with a partner, or withdrawing, or emotional warning sign, such as anger, irritability, or sadness regardless of gun ownership. Fewer than half of participants included at least one cognitive warning sign, such as rumination, “mind fog,” or indicating specific thoughts such as, “What am I doing here?” Our findings demonstrate homogeneity in the distribution and frequency of reported warning signs, when further categorized as behavioral, cognitive, emotional, or physiological, across participants when controlling for age, sex, and race among participants in our sample. In the United States, suicidal thoughts, attempts, and deaths vary between and within these groups; we hypothesized that significant differences based on these characteristics may lend insight as to discrepancies in suicide risk and deaths (1, 2).

Third, we found that participants were not likely to report both a behavioral and physiological warning sign. Based on preliminary analyses, we hypothesized that there would be measurable differences between gun owners and non-owners on the frequency and type of warning signs reported. Our results did not support this hypothesis, however, as behavioral warning signs were reported nearly as often as emotional warning signs, this may suggest that such signs may better serve to indicate imminent risk of suicide than cognitive or emotional warning signs alone (8, 25, 39). Literature on warning signs, including cognitive, behavioral, emotional, and physiological, around a suicide crisis and emotional distress may not be correlated with commonly identified risk factors (25, 40, 41) and individuals with commonly identified risk factors may not report only cognitive or emotional warning signs.

We argue that our findings yield several implications. First, we maintain that using a combination of qualitative and quantitative approaches to both study design and analysis, such as through content analysis, adds value in suicide research. Further examination using such methods will reveal opportunities for future prevention and intervention strategies, such as crisis response planning among non-clinical groups and with high-risk groups such as individuals who own or have access to firearms. Despite a lack of statistical significance in differences in self-reported warning signs across key participant characteristics, including suicidal ideation, and between gun owners and non-owners, we argue that parsing warning signs using the suicidal mode (32) between these groups may influence future identification and prevention strategies. Further, these similarities among groups may be moderated by self-reported self-management strategies when examining outcome variables related to suicide.

Several limitations must be considered as to the context and implications of these findings and results should be considered preliminarily until further research is conducted Participant CRPs were completed as part of a larger clinical trial which employed stratified sampling informed by a priori power analyses on other variables of interest, therefore, our sample size was limited to those who also fit criteria for the parent study. Participants were also not asked to report their gender identity and were asked to report their sex assigned at birth; these results are limited in their generalizability to individuals with gender and sexual minority identities. As Asarnow et al. (42) also noted, gun owners are disproportionally male and White, which was reflected as well in our sample, leading to limits in generalizability. Participation also required individuals to be 18 years or older; our findings may not be consistent with children and adolescents. CRPs were completed during one encounter without opportunities to amend responses which may improve precision of awareness and recognition of warning signs which are often revealed when the CRP is used in clinical treatment settings [BCBT-SP; (9)]. Lastly, as warning signs were coded without opportunities to clarify responses with participants, we may have categorized responses that varied from the participant's intended meaning. Future studies may account for several of these limitations by expanding the sampling frame and employing additional qualitative study designs (27).

In conclusion, this study is the first attempt to codify self-reported warning signs of emotional distress or a suicide crisis with constructs of the suicidal mode (32) and the association between various warning signs and key characteristics, such as firearm ownership and suicidal ideation among a community sample using content analysis. As many people who die by suicide are far less likely to encounter mental health care preceding death with far more encountering emergency care settings, the CRP presents as a useful tool to be implemented proactively among those presenting in emergency care, especially among gun owners. Further research is needed to demonstrate potential consistencies in the associations found between warning signs, specifically behavioral and physiological warning signs, to reduce the number of lives lost to suicide, especially by firearms.

## Data Availability Statement

The raw data supporting the conclusions of this article will be made available by the authors, without undue reservation.

## Ethics Statement

The studies involving human participants were reviewed and approved by the Ohio State University Institutional Review Board. The patients/participants provided their written informed consent to participate in this study. Written informed consent was obtained from the individual(s) for the publication of any potentially identifiable images or data included in this article.

## Author Contributions

CBa, CBr, JM, and JH contributed to conception and design of the study. JM and JH reviewed, coded, and compiled participant data. CBa performed the statistical analysis and wrote the first draft of the manuscript. CBr, AS, JH, and JM wrote sections of the manuscript. AS formatted the manuscript for submission and contributed to components of the introduction. All authors contributed to manuscript revision, read, and approved the submitted version.

## Funding

This work was supported by the National Institute of Mental Health [R61MH125759-01].

## Conflict of Interest

CBr has received grant, research, or other support from The Boeing Company. He has served as a consultant on suicide prevention and treatment for Oui Therapeutics, LLC. He receives income as the co-owner of Anduril, LLC, which conducts training workshops on crisis response planning, suicide prevention, and lethal means counseling. CRB has received payment through Anduril, LLC, for training workshops on crisis response planning. The remaining authors declare that the research was conducted in the absence of any commercial or financial relationships that could be construed as a potential conflict of interest.

## Publisher's Note

All claims expressed in this article are solely those of the authors and do not necessarily represent those of their affiliated organizations, or those of the publisher, the editors and the reviewers. Any product that may be evaluated in this article, or claim that may be made by its manufacturer, is not guaranteed or endorsed by the publisher.

## References

[1] Hedegaard H Curtin S Warner M. Suicide Mortality in the United States, 1999–2019. NCHS Data Brief, No 398. Hyattsville: National Center for Health Statistics (2021). 10.15620/cdc:101761

[2] Drapeau C, McIntosh, J,. U.S.A. Suicide: 2020 Official Final Data. [Online]. Minneapolis, MN: Suicide Awareness Voices of Education (SAVE) (2021). Available online at: https://save.org/about-suicide/suicide-statistics/ (accessed January 10, 2022).

[3] Hom MA Stanley IH Joiner TE Jr. Evaluating factors and interventions that influence help-seeking and mental health service utilization among suicidal individuals: a review of the literature. Clin Psychol Rev. (2015) 40:28–39. 10.1016/j.cpr.2015.05.006 26048165

[4] Walby FA Myhre MØ Kildahl AT. Contact with mental health services prior to suicide: a systematic review and meta-analysis. Psychiatr Serv. (2018) 69:751–9. 10.1176/appi.ps.201700475 29656710

[5] Cerel J Singleton MD Brown MM Brown SV Bush HM Brancado CJ. Emergency department visits prior to suicide and homicide: linking statewide surveillance systems. Crisis. (2016) 37:5. 10.1027/0227-5910/a000354 26620917

[6] Rudd MD. Fluid vulnerability theory: A cognitive approach to understanding the process of acute and chronic suicide risk. In Ellis TE, editor. Cognition and Suicide: Theory, Research, and Therapy. Washington, DC: American Psychological Association (2006). p. 355–8. 10.1037/11377-016

[7] Stanley B Brown GK. Safety planning intervention: a brief intervention to mitigate suicide risk. Cogn Behav Pract. (2012) 19:256–64. 10.1016/j.cbpra.2011.01.00131112330

[8] Rudd MD Berman AL Joiner TE Jr Nock MK Silverman MM Mandrusiak M. Warning signs for suicide: theory, research, and clinical applications. Suicide Life Threat Behav. (2006) 36:255–62. 10.1521/suli.2006.36.3.255 16805653

[9] Bryan CJ Rudd MD. Brief Cognitive-Behavioral Therapy for Suicide Prevention. New York, NY: Guilford Publications (2018).

[10] Rajappa K Gallagher M Miranda R. Emotion dysregulation and vulnerability to suicidal ideation and attempts. Cognit Ther Res. (2012) 36:833–9. 10.1007/s10608-011-9419-2

[11] Anestis MD Kleiman EM Lavender JM Tull MT Gratz KL. The pursuit of death versus escape from negative affect: an examination of the nature of the relationship between emotion dysregulation and both suicidal behavior and non-suicidal self-injury. Compr Psychiatry. (2014) 55:1820–30. 10.1016/j.comppsych.2014.07.007 25104613

[12] Forkmann T Scherer A Böcker M Pawelzik M Gauggel S Glaesmer H. The relation of cognitive reappraisal and expressive suppression to suicidal ideation and suicidal desire. Suicide Life Threat Behav. (2014) 44:524–36. 10.1111/sltb.12076 24494723

[13] Law KC Khazem LR Anestis MD. The role of emotion dysregulation in suicide as considered through the ideation to action framework. Curr Opin Psychol. (2015) 3:30–5. 10.1016/j.copsyc.2015.01.014

[14] Harris L Chelminski I Dalrymple K Morgan T Zimmerman M. Suicide attempts and emotion regulation in psychiatric outpatients. J Affect Disord. (2018) 232:300–4. 10.1016/j.jad.2018.02.054 29500958

[15] Lynch TR Cheavens J Morse JQ Rosenthal MZ. A model predicting suicidal ideation and hopelessness in depressed older adults: the impact of emotion inhibition and affect intensity. Aging Ment Health. (2004) 8:486–97. 10.1080/13607860412331303775 15724830

[16] Miranda R Nolen-Hoeksema S. Brooding and reflection: rumination predicts suicidal ideation at 1-year follow-up in a community sample. Behav Res Ther. (2007) 45:3088–95. 10.1016/j.brat.2007.07.015 17825248 PMC4026259

[17] Miranda R Tsypes A Gallagher M Rajappa K. Rumination and hopelessness as mediators of the relation between perceived emotion dysregulation and suicidal ideation. Cognit Ther Res. (2013) 37:786–95. 10.1007/s10608-013-9524-5

[18] Kudinova AY Owens M Burkhouse KL Barretto KM Bonanno GA Gibb BE. Differences in emotion modulation using cognitive reappraisal in individuals with and without suicidal ideation: an ERP study. Cogn Emot. (2016) 30:999–1007. 10.1080/02699931.2015.1036841 25978547

[19] Brausch AM Clapham RB Littlefield AK. Identifying specific emotion regulation deficits that associate with nonsuicidal self-injury and suicide ideation in adolescents. J Youth Adolesc. (2021) 51:556–69. 10.1007/s10964-021-01525-w 34686951 PMC9554798

[20] Wang J Sumner SA Simon TR Crosby AE Annor FB Gaylor E. Trends in the incidence and lethality of suicidal acts in the United States, 2006 to 2015. J Am Med Assoc Psychiatry. (2020) 77:684–93. 10.1001/jamapsychiatry.2020.0596 32320023 PMC7177650

[21] Osgood CE Walker EG. Motivation and language behavior: a content analysis of suicide notes. J Abnorm Soc Psychol. (1959) 59:58. 10.1037/h0047078 13664411

[22] Niederkrotenthaler T Voracek M Herberth A Till B Strauss M Etzersdorfer E. Role of media reports in completed and prevented suicide: Werther v. Papageno effects. Br J Psychiatry. (2010) 197:234–43. 10.1192/bjp.bp.109.074633 20807970

[23] Synnott J Ioannou M Coyne A Hemingway S. A content analysis of online suicide notes: attempted suicide versus attempt resulting in suicide. Suicide Life Threat Behav. (2018) 48:767–78. 10.1111/sltb.12398 28960422

[24] Krippendorff K. Content Analysis: An Introduction to Its Methodology. New York, NY: Sage Publications (2018). 10.4135/9781071878781

[25] Adler A Bush A Barg FK Weissinger G Beck AT Brown GK. A mixed methods approach to identify cognitive warning signs for suicide attempts. Arch Suicide Res. (2016) 20:528–38. 10.1080/13811118.2015.1136717 26761398

[26] Rourke L Anderson T. Validity in quantitative content analysis. Educ Technol Res Dev. (2004) 52:5–18. 10.1007/BF02504769

[27] Hjelmeland H Knizek BL. Why we need qualitative research in suicidology. Suicide Life Threat Behav. (2010) 40:74–80. 10.1521/suli.2010.40.1.74 20170263

[28] Bryan CJ Mintz J Clemans TA Burch TS Leeson B Williams S. Effect of crisis response planning on patient mood and clinician decision making: a clinical trial with suicidal U.S. soldiers. Psychiatr Serv. (2018) 69:108–111. 10.1176/appi.ps.201700157 28967323

[29] Stanley B Brown GK Brenner LA Galfalvy HC Currier GW Knox KL. Comparison of the Safety planning intervention with follow-up vs usual care of suicidal patients treated in the emergency department. J Am Med Assoc Psychiatry. (2018) 75:894. 10.1001/jamapsychiatry.2018.1776 29998307 PMC6142908

[30] Miller IW Camargo CA Arias SA Sullivan AF Allen MH Goldstein AB. Suicide prevention in an emergency department population: the ED-SAFE study. JAMA Psychiatry. (2017) 74:563–70. 10.1001/jamapsychiatry.2017.0678 28456130 PMC5539839

[31] Rozek DC Keane C Sippel LM Stein JY Rollo-Carlson C Bryan CJ. Short-term effects of crisis response planning on optimism in a US Army sample. Early Interv Psychiatry. (2019) 13:682–5. 10.1111/eip.12699 29943518

[32] Rudd MD. The suicidal mode: a cognitive-behavioral model of suicidality. Suicide LifeThreat Behav. (2000) 30:18–33. 10.1111/j.1943-278X.2000.tb01062.x 10782716

[33] Hsieh H-F Shannon SE. Three approaches to qualitative content analysis. Qual Health Res. (2005) 15:1277–88. 10.1177/1049732305276687 16204405

[34] Fox KR Harris JA Wang SB Millner AJ Deming CA Nock MK. Self-injurious thoughts and behaviors interview—revised: development, reliability, and validity. Psychol Assess. (2020) 32:677. 10.1037/pas0000819 32324021

[35] Krippendorff K. Estimating the reliability, systematic error and random error of interval data. Educ Psychol Meas. (1970) 1:61–70. 10.1177/001316447003000105

[36] Hayes AF Krippendorff K. Answering the call for a standard reliability measure for coding data. Commun Methods Meas. (2007) 1:77–89. 10.1080/19312450709336664

[37] StataCorp. (2019). Stata Statistical Software: Release 16. College Station TX: StataCorp, LP.

[38] McFadden D. Conditional logit analysis of qualitative choice behavior. In: Zarembka P, editor. Frontiers in Econometrics. New York, NY: Academic Press (1973). p. 105–42.

[39] Franklin JC Ribeiro JD Fox KR Bentley KH Kleiman EM Huang X. Risk factors for suicidal thoughts and behaviors: a meta-analysis of 50 years of research. Psychol Bull. (2017) 143:187. 10.1037/bul0000084 27841450

[40] Bryan CJ Rudd MD. Advances in the assessment of suicide risk. J Clin Psychol. (2006) 62:185–200. 10.1002/jclp.20222 16342288

[41] Klonsky ED May AM. Differentiating suicide attempters from suicide ideators: a critical frontier for suicidology research. Suicide Life Threat Behav. (2014) 44:1–5. 10.1111/sltb.12068 24313594

[42] Asarnow JR Zullo L Ernestus SM Venables CW Goldston DB Tunno AM. “Lock and Protect”: development of a digital decision aid to support lethal means counseling in parents of suicidal youth. Front Psychiatry. (2021) 12. 10.3389/fpsyt.2021.736236 PMC8528190 34690841

